# Human Capital and Labour Market Resilience: A Regional Analysis for Portugal

**DOI:** 10.1007/s12061-022-09465-z

**Published:** 2022-06-02

**Authors:** Marta Simões, João Sousa Andrade, Adelaide Duarte

**Affiliations:** Univ Coimbra, CeBER, Faculty of Economics, Av Dias da Silva 165, 3004-512 Coimbra, Portugal

**Keywords:** Human Capital, Education, Employment, Resilience, NUTS2, Portugal

## Abstract

This paper investigates labour market resilience for seven Portuguese NUTS-2 regions over the period 1995–2018 detailing its relationship with levels of education and highlighting the period following the 2007–08 financial and economic crisis. We define resilience as the ability of regional employment to recover from a recessionary shock over an entire business cycle. Our results point to the existence of labour market resilience to the different business cycles for the different regions in terms of total hours worked. The same conclusion applies to employment of workers with different levels of educational attainment, low, medium and high, defined according to the highest level of education completed by employees. Investigating in more detail the potential differentiated impact of the Portuguese Great Recession (PGR), covering the period after the 2007–08 crisis, our findings suggest however no resilience in terms of total hours worked and employment of workers with low levels of education, corresponding so far to a situation of jobless economic recovery. The conclusions are mixed for employment of workers with medium levels of education, while we found evidence of labour market resilience to the PGR for employment of workers with high levels of education. The strong negative impact of the PGR at the economic level thus seems to have hindered labour market resilience for employees and regions less endowed with human capital.

## Introduction

The adverse shock that resulted in the 2007–08 financial and economic crisis and the Great Recession that ensued generated an unprecedented interest in economic resilience.^1^ The Brittanica dictionary defines resilience as ‘the ability to become strong, healthy, or successful again after something bad happens.’ (https://www.britannica.com/dictionary/resilience) Resilience is now routinely addressed in economics research with the main aim of preparing economies to deal with the consequences of disturbances, with a wealth of studies focusing on the regional dimension of resilience from the perspective of output and employment, Simmie and Martin (2010); Fingleton et al. (2012); Martin (2012); Doran and Fingleton (2016); Martin et al. (2016); Sensier et al. (2016); Angulo et al. (2018); Di Caro (2018); Faggian et al. (2018); Lapuh (2018); Alpek and Tésits (2019); Cuéllar-Martín et al. (2019); Ringwood et al. (2019). However, the concept of regional resilience has been used with different meanings as stressed by Martin (2012) and Martin and Sunley (2015) that distinguish between engineering (the ability to return to the initial path following a shock), ecological (shocks may affect the path of the economy in a permanent way) and adaptative (the capacity of an economy to adapt successfully to disturbances by renewing itself) notions of resilience involving at least one of four elements that characterise the response to a shock, resistance, recovery, reorientation and renewal.

This study contributes to two strands of literature on economic resilience from the perspective of the labour market, on the characterization of regional resilience to economic crises and on the analysis of the determinants of resilience, Giannakis and Bruggeman (2017); Di Caro and Fratesi (2018); Fratesi and Perucca (2018); Gutiérrez Posada et al. (2018); Cappelli et al. (2020). We depart from previous definitions of resilience by considering that a region is resilient, in the sense that it recovers from shocks, if the latter have no impact on employment over the varied business cycles that characterize the period under analysis. In this way we overcome the need to define measures of resistance and recovery to a particular shock, with different measures and shocks potentially resulting in different and less comparable results since resilience according to one measure need not imply resilience according to another. Also, the regional labour market may be resilient (recover from) to one shock but not to another. With our approach we are thus able to give a broader picture of resilience since we investigate recovery over the whole period under analysis when several shocks hit the regional economy. In any case, since the severity of certain shocks may change the resilience profile of the regional labour market, we investigate whether the response to the 2007–08 shock is different. To be clear also, we do not analyse the intensity of the response to a shock nor the speed of recovery. Moreover, we depart from previous analyses by focusing on resilience in the private sector labour market, more flexible than the public-sector labour market and thus more likely to adapt to shocks. Education, the driver of resilience that we highlight since it is one of the factors that come out most clearly in previous literature as impacting on employment resilience, is also more likely to influence resilience in the private sector labour market (Martin & Gardiner, 2019). Additionally, we deal with regional labour markets resilience from the perspective of hours worked, which can have important implications for regional growth and development (Hart, 2017).

The objective of this work is threefold: i) investigate labour market resilience in the context of the seven Portuguese NUTS-2 regions over the period 1995–2018 detailing its relationship with levels of education and focusing on the effects of the 2007–08 economic and financial crisis and the sovereign debt crisis that ensued, a period that we dub as the Portuguese Great Recession (PGR); ii) cover different recessions and shock-recovery periods to detect changes in importance of the regional attribute highlighted, levels of education, to the explanation of the potentially different regional resilience paths; and iii) investigate possible episodes of jobless recovery. Our approach aims at answering the following more general questions: do regions differ in their resilience to shocks, that is in their ability to recover from such disruptions? Are the effects of shock merely temporary, irrespective of the time that recovery takes? Do severe disruptions have differentiated effects? Is resilience related to human capital availability?

Portugal is one of the European Union (EU) member states most severely hit by the 2007–08 financial crisis resulting in the need for fiscal consolidation. The Portuguese sovereign debt crisis called for a bailout from the International Monetary Fund (IMF), the European Central Bank (ECB) and the European Commission (EC) that lasted from May 2011 to June 2104 and implied the implementation of severe austerity measures. According to PORDATA database, in 2009 Portuguese real GDP dropped 3.12%, and in 2012 the drop was stronger, 4.06%. During the same period the unemployment rate steadily increased and in 2013 it reached 16.2%. Portugal also compares poorly with the average EU member state in terms of educational attainment: Eurostat data for the year 2019 shows that 47.6% of the Portuguese 15–64 year-olds had less than lower secondary education while the EU-28 average was 24.9%. This is particularly worrisome since education, the main source of human capital accumulation, is an important determinant of economic resilience, long run growth and various social outcomes (Benos & Zotou, 2014; Ramos et al., 2012; Panori & Psycharis, 2019; Salas-Velasco et al., 2021). At the regional level Portugal is an unequal country. Four of the seven Portuguese NUTS2 regions are classified by the EC as less developed (*Norte, Centro, Alentejo* and *Açores*), the *Algarve* is considered a transition region and only *Lisboa* and *Madeira* are classified as more developed regions. Alexandre et al. (2021) and Correia and Alves (2017) describe varied dynamic patterns in the Portuguese regions both in terms of output and employment and the Global Labour Resilience Index (GLRI) 2020 (Whiteshield Partners, 2020) highlights education and skills as a priority for Portugal to increase labour market resilience emphasising the need for analyses at the regional level.

We test for resilience applying an adapted version of the seemingly unrelated regression (SUR) methodology proposed by Fingleton et al. (2012) that include as explanatory variables of employment growth different dummies corresponding to specific recession periods in 12 UK NUTS-1 regions identified for the period 1971–2010 and variables capturing the change in employment growth rate in post-recession periods. Our SUR model consists of seven equations, one for each Portuguese NUTS-2 region observed over 24 years. The dependent variable is a measure of employment and the explanatory variables of interest are the two dummies defined according to the sign (positive or negative) of the regional output gap. We define resilience as the ability of employment to recover from the impact of shocks occurring during the whole period under analysis, which corresponds to the situation when the null hypothesis of equality of the coefficients of the dummies cannot be rejected. Additionally, we investigate in more detail the resilience of the regional labour markets to the PGR since severe disruptions may permanently alter the structure and trajectory of the affected regional economy (Martin & Gardiner, 2019). We do this through the consideration of regional time dummies for the period 2009–2018 depending on the sign of the output gap and testing the equality of the coefficients associated with the dummies for recession vs. expansion years.

The remainder of this work is set out as follows: in the next section we give an overview of the literature on regional labour market resilience and its relationship with human capital; Sect. 3 contains the methodology and data description; Sect. 4 presents and discusses the results; and Sect. 5 concludes.

## An overview of the Literature on Regional Labour Market Resilience

Extant literature that investigates economic resilience at the regional level shows that regions within a country may not be equally able to face adverse shocks, notably in terms of the labour market and associated levels of employment. Given the controversies on the definition of resilience and following the Great Recession initiated in 2007–08, there has been a renewed interest in measuring and describing regional resilience and more recent studies are also concerned with the identification of the determinants of regional resilience, see e.g. Simmie and Martin (2010); Fingleton et al. (2012); Martin (2012); Dubé and PolèSe (2016); Sensier and Artis (2016); Sensier et al. (2016); Angulo et al. (2018); Di Caro (2018); Faggian et al. (2018); Lapuh (2018); Ringwood et al. (2019).

Indeed, the concept of resilience under analysis varies across studies. The often cited works of Martin (2012) and Martin and Sunley (2015) provide the fundamental definitions of resilience that have been used in the investigation of this phenomenon at the regional level, engineering, ecological and adaptative resilience.

Engineering resilience refers to the ability of a system to withstand and recover from shocks or disturbances. This type of resilience has been associated with the plucking model of business fluctuations (Friedman, 1993), the neoclassical growth model according to which shocks have only transitory growth effects and more generally to real business cycle theory or endogenous growth models that emphasize the differences between short and long-run dynamics relative to the equilibrium situation, Fingleton et al. (2012).

Ecological resilience is related to the capacity of a region to keep functioning within the same state or equilibrium in the presence of a shock before changing to a new equilibrium. From an economic perspective, this might imply the existence of multiple equilibria or steady states so that the shock can move the region to a different steady state. In this case, the notion of hysteresis applies, i.e. the shock may have a permanent effect on the regional equilibrium level and/or growth path, Fingleton et al. (2012); Ringwood et al. (2019).

Finally, adaptative resilience is concerned with the ability of a region to adapt and renew itself in response to a shock. This understanding of resilience aligns well with the evolutionary economics perspective and the idea that regional economies are continually changing and adapting, rejecting the ‘equilibrist’ approach, Reggiani et al. (2002); Simmie and Martin (2010); Diodato and Weterings (2014).

From these main types of resilience, Martin (2012) identifies four interrelated dimensions of resilience: resistance—the sensitivity to disturbances of a regional economy; recovery—the speed and extent of regional recovery after a perturbation; re-orientation—the extent of structural changes in the regional economy following a shock; and renewal—the degree of renewal or resumption of the growth trajectory that characterised the regional economy before the disturbance.

The bulk of the more recent literature on regional labour markets resilience has dealt with recovery from the economic and financial crisis that started in 2007–08, see e.g. Doran and Fingleton (2016); Dubé and PolèSe (2016); Faggian et al. (2018); Lapuh (2018); Ringwood et al. (2019), Gong et al. (2020). However, the regional economy is constantly being hit by shocks and thus a broader picture of resilience involves looking at recovery for more extended periods that cover several shocks. This approach is scarcer in the literature. Fingleton et al. (2012) and Martin and Gardiner (2019) constitute examples of the few studies that test for resilience to different adverse shocks, concentrating in the specific cases of what they define as major recessions that however are not region-specific but defined at the national level.

Fingleton et al. (2012) approach resilience from the perspective of engineering resilience using quarterly employment data for 12 UK NUTS-1 regions observed over the period 1971–2010. The authors estimate a SUR model to test resilience of regional employment to the four recessionary shocks that they define for their sample based on the dating of the business cycle for the national economy. The former are included in the regressions as dummy variables, together with the change in employment growth rate in the different post-recession periods. The evidence found shows that regions differ in terms of their resistance to shocks (the exception is the 2008–2009 recession) but not in their recovery employment growth. For a similar period as the one analysed by Fingleton et al. (2012) but focusing on the 85 British largest cities, Martin and Gardiner (2019) investigate resistance to and recovery from the four major recessions that occurred during the period 1971 to 2015 in the UK by computing resistance and recovery indices. They conclude that there was significant variation across British cities in both their resistance to and recoverability from the four recessions, with resilience of cities itself varying over time. More specifically, whereas in the first two recessions the more resistant a city the faster its recovery, in the second two recessions this relationship disappeared, showing at most the less resistant a city the faster its recovery.

The differences in resilience found across regions and over time have spurred research into its causes (Fingleton et al., 2012; Martin & Gardiner, 2019). Within the analysis of the drivers of labour market resilience, human capital in the form of educational attainment is present and confirmed as a crucial determinant of resilience by many previous studies, which use varied proxies but mostly referring to more advanced schooling levels. For instance, Di Caro (2017) uses quarterly regional employment data over the period 1992–2012 for the 20 Italian NUTS2 regions to obtain OLS regression results with measures of engineering and ecological resilience as dependent variables that indicate that industrial diversification, high export ability, low financial constraints and rich endowments of human (average years of schooling) and social capital enhance regional resilience.

Giannakis and Bruggeman (2017) investigate the pre-crisis (2002–2007) determinants of resilience, assessed based on employment changes during 2008–2013, to economic crisis across 268 NUTS2 regions of the EU-28 using a multilevel logistic regression model. From the 15 predictor variables used, education, measured as the share of workforce aged 25–64 years with upper secondary, post-secondary and tertiary education is found to be the most important determinant of employment resilience. The authors highlight the fact that “All 7 Portuguese regions were among the 10 regions with the lowest shares of workforce with higher education across EU-28, ranging from 42% (PT17 – *Área Metropolitana de Lisboa*) to 21% (PT20 – *Região Autónoma dos Açores*).” (p. 1405).

Another country specific study is that by Kitsos and Bishop (2018) with a focus on the impact of a number of factors on employment resilience in Great Britain following the 2007–08 shock. Three variables are used to represent human capital: the shares of skilled and unskilled workers and employee training rates. Cross-section OLS findings confirm a positive role for the share of skilled workers and a younger population, while there was a lack of consistently significant results for industrial structure, diversity and entrepreneurship.

Cappelli et al. (2020) provide a more encompassing analysis, again for the EU NUTS2 regions, but looking at the resistance of unemployment following the 2008 crisis and the role played by technological and human capital. The authors regress their measure of unemployment resistance over the period 2008–16 on a set of explanatory variables that include human capital (the percentage of the population aged 25–64 with tertiary education) using OLS. The results indicate that human capital alone is not enough to enhance unemployment resistance, although a positive effect appears when human capital is interacted with an indicator of technological resistance.

From the sole perspective of Portugal, previous studies of resilience at the regional level are scarce. Hennebry (2020) examines the determinants of economic resilience focusing on 16 Portuguese NUTS3 rural regions after the 2008–09 crisis. From the bivariate analysis carried out applying the Pearson correlation coefficient, the author concludes that employment resilience is highly negatively associated with the number of patents, reliance on tourism, employment in manufacturing, crime and higher voter turnout, while presenting a positive association with the median age of the population and employment in agriculture. The correlation with the share of the labour force with tertiary education found was positive but not statistically significant.

This study defines resilience as the capacity of regional employment to recover after a recession paying attention to employment distributions across workers with different endowments of human capital/education covering low, medium and higher levels of schooling, since resilience to the crisis may differ according to these different endowments. The methods employed in the literature to examine resilience concentrate on recovery from a specific shock, implying its identification in time and its measurement. Besides considering the entire distribution of human capital, we also aim to contribute to the literature by applying a SUR methodology that allows detecting resilience to shocks in general, based on standard definitions and measures of the business cycle (the output gap) that endogenously define the relevant sub-periods for the analysis and thus the examination of broader patterns of resilience. Different from previous studies also our dating of the business cycles is region specific not imposing the same business cycles as those for the national economy. In summary, our approach to resilience acknowledges regions’ capacity to recover after an (general) adverse shock and the role of human capital availability analysing the ‘trend’ in employment resilience and acknowledging that results are dependent on the existence of certain factors.

## Data and Methods

We build a regional database for Portugal with annual data for the seven Portuguese NUTS2 regions spanning over the period 1995–2018. We collected data from three primary statistical sources, Eurostat (Regional Economic Accounts (reg_eco10)), the National Statistics Office, INE (Regional Accounts) and *Quadros de Pessoal* – *QP* (Personnel Records). *QP* is a linked employer-employee dataset gathered annually by the Portuguese Ministry of Labour since 1985 and covering all establishments having at least one wage earner. The dataset contains information on gender, age, education, monthly wages, and hours worked, among others, for each employee (excluding civil servants, self-employed and household employees). We computed the following variables: the (real GDP) output gap, GAP; the number of employees by levels of schooling with, respectively, less than 9 years of schooling, G1, at least 9 but less than 12 years of schooling, G2, and with more than 12 years of schooling, G3; and total hours worked (regular plus overtime hours), HT; and different dummy variables that will be identified in the next section.

The choice of period was dictated by data availability at the regional level and methodological issues. Empirical methods such as the SUR method are very demanding in terms of number of observations. To use the highest number of observations available, we consider the first year for which we have detailed data at the regional level from INE, 1995, and as end year 2018, the most recent year for which we have data from *QP*. Notice however that regional data availability from 1995 onwards precludes resilience comparisons between the PGR and the two other similar negative shocks that hit the Portuguese economy and also involved IMF interventions (1978–79 and 1983–84).

The seven Portuguese regions are unequal, namely in terms of wealth creation and population. *Lisboa*, *Norte* and *Centro* are jointly responsible for the creation of 84.6% (1995–98) and 84.4% (2015–2018) of the country's wealth (GDP at constant 2015 prices) and concentrate 83.9% of the population in both periods. The joint contribution of the two remaining continental regions, *Algarve* and *Alentejo*, is 11.4% and 11%, respectively, and their population corresponds to 11.3% of the total population. The regions of *Madeira* and *Açores* contribute jointly with 4.2% and 4.6% to the country's GDP and concentrate 4.8% and 4.9% of total population. The regional real GDP ranking (in descending order) remains unchanged between the two periods: *Lisboa, Norte, Centro, Alentejo, Algarve, Madeira* and *Açores*; in the case of population, the ranking remains unchanged for the six most populated regions: *Norte, Lisboa, Centro, Alentejo, Algarve* and *Madeira*, while *Açores*, previously *ex aequo* with *Madeira*, drops from the sixth to the seventh position.

We disaggregate employment according to schooling levels, low (G1), medium (G2) and high (G3). Employment corresponds to the number of employees engaged in the private sector. Table 1 contains data on the share of employees with different schooling levels for each region and at the country level. We observe that all the NUTS2 regions follow a similar path concerning the regional composition of employment by levels of schooling with the share of employees with less than 9 years of schooling (G1) decreasing and the shares of employees with at least 9 and less than 12 years of schooling (G2) and with more than twelve years of schooling (G3) increasing over time. *Lisboa* always leads in terms of the contribution of G1 and G2 to employment. In 2015–18, the regional ranking in terms of the contribution of G3 to employment is, in descending order: *Lisboa*, *Norte*, *Centro*, *Madeira*, *Alentejo* and *Algarve* and *Açores* in the same bottom position. Educational policies together with an increase in demand for more educated workers help to explain the evolution of the composition of employment.

**Table 1:** Share of employment by levels of schooling (%), 1995–2018

	Employees with less than 9 years of schooling		Employees with at least 9 and less than 12 years of schooling		Employees with more than 12 years of schooling	
	1995–98	2015–18	1995–98	2015–18	1995–98	2015–18
*Alentejo*	82.9	58.3	11.8	25.7	5.3	16.1
*Algarve*	79.8	56.4	15.4	28.5	4.8	15.1
*Açores*	83.4	62.7	12.0	23.3	4.7	14.0
*Centro*	83.9	57.9	10.8	24.8	5.3	17.3
*Lisboa*	69.8	41.3	20.5	32.0	9.7	26.6
*Madeira*	83.1	56.6	14.4	27.2	2.6	16.3
*Norte*	85.2	58.6	10.2	24.0	4.6	17.4
Portugal	79.1	52.1	14.3	27.4	6.5	20.5

For each region and period, the horizontal sum is 100.0%. Rounding errors can explain slight deviations

Sources: authors’ calculations based on data from *Quadros de Pessoal*

**Fig. 1:**
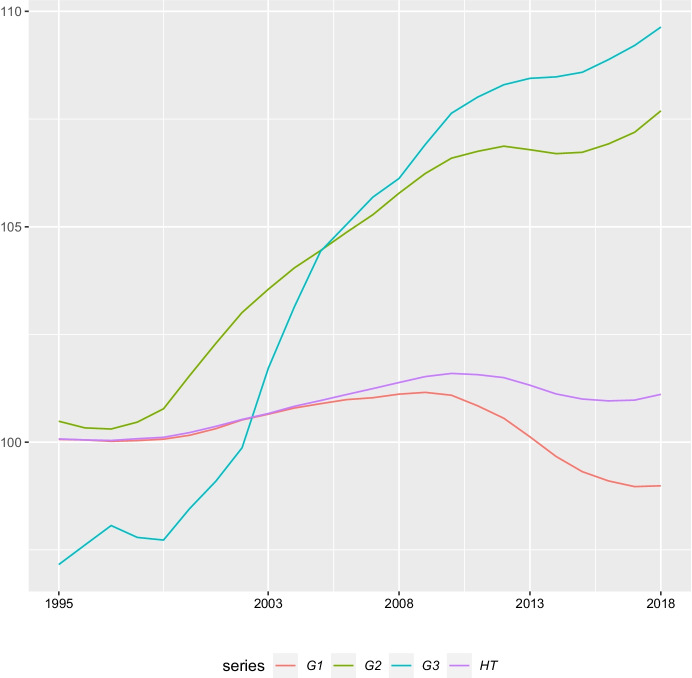
Employment by levels of schooling (logs) at the country level, 1995–2018. Source: authors’ own calculations using data from *Quadros de Pessoal*

Figure 1 is informative about the evolution of employment composition by levels of schooling at the country level: the relative importance of G3 and G2 has increased in almost all the years and the opposite applies to G1. Total hours worked progressed at a slower pace than G2 and G3, a consequence of the decrease in G1. The sub-period 2008–2013 comprises the economic and financial crisis and the Portuguese sovereign debt crisis. Due to the crisis Portugal was under a stabilization/economic adjustment programme. With the austerity measures implemented, employment decreased sharply. If we compute the percentage change of the employment variables in the year when they recorded the minimum value relative to the respective value in the year 2008, when the PGR started, we get a drop of -33% for G1, -9% for G2, and -4% for G3 and -20% for HT. In slightly different terms, in 2012 G1 corresponds to only 72% of the respective value in 2008; in that same year G2 corresponds to 91% of the respective 2008 value; in 2010 G3 corresponds to 96% of the respective 2008 value, and, finally, in 2013 this value for HT is 82%. Indeed, the Portuguese sovereign debt crisis was quite severe, with the rate of unemployment reaching 9.4% in 2009 and 16.2% in 2013. An emigration wave comprising 495,504 individuals, including the younger and more educated ones followed (Pereira, 2011).

We need to compute, at the regional and national levels, the cyclical component of output (GAP), according to which when the output gap is negative (positive) the economy is in a recession (expansion) phase. For this purpose, we first calculated real GDP (2015 prices) using the annual growth rates of regional gross value added (GVA) at basic prices by NUTS2 with 100 = 2015 (code: NAMA_10R_2GVAGR). The former were applied to the values of GDP at current prices for the base year 2015 (code: NAMA_10R_2GDP). The series for real GDP were next filtered using the H-P filter (Hodrick & Prescott, 1997) in order to obtain the GAP i.e. the H-P cyclical component of the series, with end-points bias correction (Mise et al., 2003) using ARIMA models with an optimal parameter search proposed by Hyndman and Khandakar (2008). Finally, we applied the H-P filter to the augmented series (Balcilar, 2019). We set the value of lambda at 6.25 as suggested by Ravn and Uhlig (2002). Portuguese business cycle series computed in this way matches the dates for the turning points and associated business cycles proposed by the Portuguese Business Cycle Dating Committee using quarterly data (CDCEFFMS, 2020). After obtaining the values for the output gap from the augmented series we eliminated the first three and the last three annual observations.

**Fig. 2:**
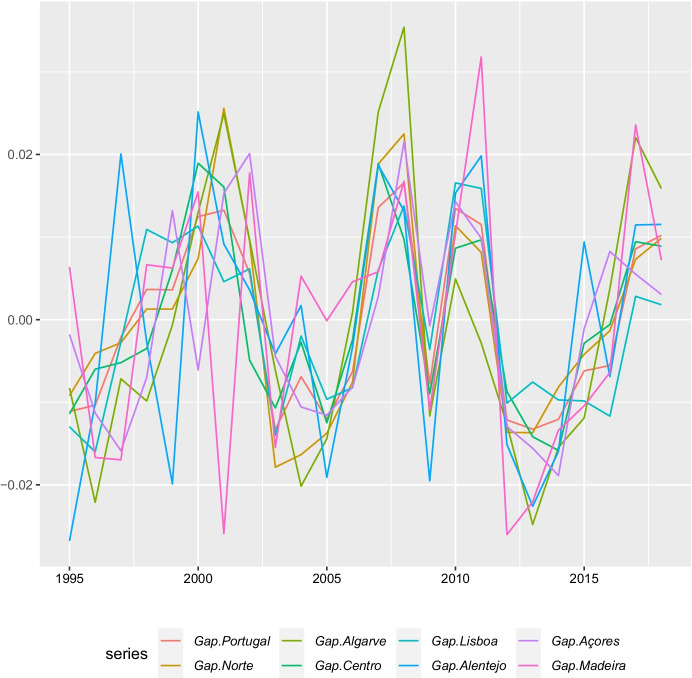
Output gaps at the regional and country level 1995–2018. Sources: Authors’ own calculations using Eurostat and INE data

Figure 2 contains our series of output gaps for the different regions and at the country level. Considering that we measure the business cycle from trough to trough, it is possible to see that Portugal presents three business cycles (1995–2003; 2003–2009; 2009–2013), and the same applies to *Açores* (1997–2000; 2000–2005; 2005–20,014), *Algarve* (1996–2004; 2004–2009; 2009–2013), *Centro* (1995–2005; 2005–2009; 2009–2014), *Lisboa* (1996–2003; 2003–2009; 2009–2016), and *Norte* (1995–2003; 2003–2009 and 2009–2013). The average duration of the business cycle varies between 5.7 years (*Açores* and *Algarve*) and 6.7 years (*Lisboa*) and the average amplitude of the absolute value of the trough varies between 0.011 (*Lisboa*) and 0.052 (*Norte*). *Alentejo* shows a different business cycle pattern with a higher number of business cycles, five (1995–1999; 1999–2005; 2005–2009; 2009–2013; 2013–2016), with an average duration of 4.2 years and an average amplitude of 0.191. The business cycles do not overlap showing different start and end dates, duration and amplitude, although most of the regions exhibit the same number of business cycles. These findings imply that we should work with the regional (not the national) output gaps to avoid mismeasurement of employment resilience.

As noted in the literature review section, several economic models have been used to investigate the multiple dimensions of economic resilience, in turn tested applying a variety of empirical methods including (descriptive) statistical data analysis (see e.g. Hennebry, 2020) and more often econometric analyses such as the SUR model (see e.g. Fingleton et al., 2012), the Probit model (see e.g. Doran & Fingleton, 2016) and the multilevel logistic regression model (see e.g. Giannakis & Bruggeman, 2017) that test for specific dimensions of resilience.

To test for resilience in the Portuguese regional labour markets we apply an adapted version of the SUR methodology proposed by Fingleton et al. (2012) using different proxies for resilience and applying different tests to relate the estimated coefficients. The SUR methodology has the advantage of not requiring the identification of a spatial linkage matrix, which the authors consider to be very difficult to know a priori. With SUR “(…) spatial effects come through the unobserved error component of the model (…)” (Fingleton et al., 2012: p.120). Spatial methods are also criticized on the grounds of the constancy attributed to the weights used to translate regional dependencies. To avoid the over-parameterisation problem in Fingleton et al. (2012), we opt for a common identification of each of the phases (expansion or recession) for all business cycles instead of including different dummy variables for each phase of each cycle. We also take advantage of the SUR model to test for the rejection of the null hypothesis of statistical significance of the variables in our estimations to select the appropriate model for each of the Portuguese NUTS 2 regions. Moreover, we use the ADL (augmented distribute lags) methodology to avoid spurious results due to the presence of a unit root in the series, which is the case for the employment series (results available from the authors). We thus consider the dependent variable in logs and include lagged values as explanatory variables.

To be clear, we define an employment regression for each region and test for labour market resilience based on the identification of regional business cycles for the period 1995–2018. We consider two models, A and B. Model A assesses regional employment resilience over the business cycles covering the whole period under analysis attributing the same importance to different business cycles. Model B additionally isolates the differentiated effects of the PGR on resilience. Model B thus includes the same explanatory variables as model A and some additional time dummy variables. For each of the two models we define four variants according to the four different proxies for regional labour used.

Model A is given by Eq. (1):

1 $${Y}_{it}= {\lambda }_{i }{Y}_{i,t-1}+{\beta }_{i}{GAP}_{it}+{\gamma }_{i}{HC}_{it}+{\alpha }_{i}^{(+)}{DGAP}_{it}^{(+)}+ {\alpha }_{i}^{(-)}{DGAP}_{it}^{(-)}++{\mu }_{it}$$

*i* = *Alentejo, Algarve, Açores, Centro, Lisboa, Madeira, Norte*; *t* = 1995,…., 2018; *N* = 7 and *T* = 24.

where *Y*_*i*_ denotes different measures of labour in region *i* at time *t* (in logs) considered one at a time and corresponding to either HT, G1, G2 or G3. HT is considered because firms can respond to shocks by hiring, not hiring, or firing workers, but also, in principle, by changing the hours worked by their employees. Although labour market resilience to shocks is usually analysed in terms of employment, ignoring hours worked can be misleading since the latter can, in principle, be altered faster and in a more flexible and less costly way. Additionally, different levels of employment and hours worked can have implications for assessing the labour productivity effects of shocks. ${\lambda }_{i}$ is the coefficient of the lagged dependent variable for region *i*.

The explanatory variables are *GAP*_*it*_, the output gap (in logs); *HC*_*it*_, human capital, calculated as average years of schooling of the employees (in logs) based on the number of employees that completed the different schooling levels defined in *QP* and the duration in years of each of these schooling levels according to the Portuguese education system; and two regional dummy variables, one for positive values of the output gap,${DGAP}_{it}^{(+)}$, that takes the value 1 in the years when the output gap is positive and 0 otherwise, and one for the negative values,${DGAP}_{it}^{(-)}$, that takes the value 1 in the years when the output gap is negative and 0 otherwise.${\gamma }_{i}$,${\beta }_{i}$, ${\alpha }_{i}^{(+)}$ and ${\alpha }_{i}^{(-)}$ are the coefficients of the different explanatory variables for each region *i*. Finally, ${\mu }_{it}$ denotes the error term.

The two dummy variables allow us to assess labour market resilience to shocks considering the different business cycles over the period 1995–2018. We consider that the labour market is resilient when the null hypothesis of equality of the coefficients of ${DGAP}_{it}^{(+)}$ and ${DGAP}_{it}^{(-)}$ is not rejected. This corresponds to a situation when employment recovers (completely) from the impact of shocks over the entire period irrespective of the intensity of and response to each specific shock. We additionally include as explanatory variable the output gap, *GAP *(*L.GDP-L.GDPtrend*), because the magnitude of the output gap varies from region to region, and human capital, *HC* (average years of schooling of employees). We expect the estimated coefficient of *GAP* to capture the amplifying (dampening) effect of the business cycle on the dynamics of employment. Including *GAP* implies that we obtain symmetric responses of employment to fluctuations through this variable and in this way the gap dummy variables capture the asymmetric responses to the business cycle.

We expect a positive sign for the estimated coefficient of *GAP* since employment is considered pro-cyclical. As for the expected sign of the estimated coefficient for the association between *HC* and employment it depends on the type of employment. Higher human capital availability at the regional level is indicative of a productive specialization pattern based on a greater demand for more qualified workers, and so we expect a negative sign for the respective estimated coefficient when the dependent variable is G1, indeterminate for the case of G2 and a positive association with G3. Note that we also considered in our regressions other control variables such as the regional inflation rate, the regional real GDP growth rate, and a trend variable, as potential determinants of the dynamics of regional employment. We retained human capital only (besides *GAP*) based on goodness of fit measures for these different regressions. These results are available from the authors.

Model B is given by Eq. (2):

2 $${Y}_{it}= {\lambda }_{i }{Y}_{i,t-1}+{\beta }_{i}{GAP}_{it}+{\gamma }_{i}{HC}_{it}+{\alpha }_{i}^{(+)}{DGAP}_{it}^{(+)}+ {\alpha }_{i}^{(-)}{DGAP}_{it}^{(-)}+\sum_{h=2009}^{2013}{d}_{ih }{S}_{i,h,t}+\sum_{h=2014}^{2018}{d}_{ih }{R}_{i,h,t}+{\varepsilon }_{it}$$

Model B introduces additional explanatory variables to measure the specific effects of the PGR on employment corresponding to different regional time/year dummy variables from 2009 to 2018, identified as either slump (S) or recovery (R) dummies depending on the sign of the output GAP for Portugal (negative for the years 2009 to 2013; positive for the years 2014–2018) and also on the results of the tests for the sum of the respective estimated coefficients irrespective of their classification into S or R. Regional employment resilience to the PGR corresponds to the situation when the null hypothesis that the sum of the coefficients of the different S and R dummy variables is equal to zero is not rejected. In this situation the negative effects of the PGR on employment vanish over the time period covered by this particular episode.

## Results and Discussion

We start by presenting and analysing the results for the four variants of model A. As can be seen from the inspection of the results presented in Table 2, the four variants of model A present low values of the respective residual standard error (RSE), indicative of their goodness of fit, although the RSE for model A4 is always slightly higher in all regions. Concerning the results with model A1, the estimated coefficient for GAP is positive and statistically significant for the regions *Alentejo* and *Centro*, and also positive, although not statistically significant for *Algarve*, *Lisboa*, *Madeira* and *Norte*. But in *Açores* it is negative. However, in the former and in all the remaining cases where it is negative if we multiply the minimum and the maximum values of the different regional output gaps by the respective estimated coefficient they are always lower than the values of the estimated coefficients of *DGAP*^*(*+*)*^ and *DGAP*^*(−)*^. We can thus conclude that the behaviour of employment proxied by total hours worked is mainly determined by *DGAP*^*(*+*)*^ and *DGAP*^*(−)*^. Additionally, it is not possible to reject the null hypothesis that *DGAP*^*(*+*)*^ and *DGAP*^*(−)*^ estimates are equal based on the results of a Wald test using the Chi-Square statistic. Hence, we conclude that there is labour market resilience in the different NUTS2 regions because for the period under analysis the employment impact of the negative output gaps is compensated by the impact of the positive output gaps.

**Table 2:** SUR Results for the regional employment models A

	Model A1		Model A2		Model A3		Model A4	
*Dependent variable*	HT		G1		G2		G3	
*DGAP*^*(−)*^*.Alentejo*	3.2355	***	2.0063	***	1.0798	***	1.99066	***
*DGAP*^*(*+*)*^*.Alentejo*	3.2254	***	1.9810	***	1.0803	***	2.03327	***
*GAP.Alentejo*	1.1469	**	1.4110	***	0.5327		0.00861	
*L.Alentejo_HC*			-0.1156	***	0.0594		0.70693	***
*L.Alentejo_Y_1*	0.8085	***	0.8483	***	0.8860	***	0.63268	***
*RSE*	0.04		0.036		0.05		0.146	
*DGAP*^*(−)*^*.Algarve*	2.5622	***	2.2005	***	0.87249	**	1.3435	***
*DGAP*^*(*+*)*^*.Algarve*	2.5719	***	2.2175	***	0.89414	**	1.3376	***
*GAP.Algarve*	0.5515		0.4192		0.22053		3.1398	
*L.Algar_HC*			-0.0716		0.00405		0.9412	***
*L.Algar_Y_1*	0.8453	***	0.8161	***	0.91646	***	0.6366	***
*RSE*	0.065		0.06		0.066		0.181	
*DGAP*^*(−)*^*.Açores*	1.7051	*	0.9177		0.4781		2.055	***
*DGAP*^*(*+*)*^*.Açores*	1.7137	*	0.9659		0.5382		2.104	***
*GAP.Açores*	-1.0776		-1.5031		-2.3449	**	1.265	
*L. Açores_HC*			-0.1300	**	-0.1297		1.346	***
*L. Açores_Y_1*	0.8922	***	0.9371		0.9805	***	0.399	***
*RSE*	0.055		0.049		0.045		0.177	
*DGAP*^*(−)*^*.Centro*	3.3202	***	1.8917	***	1.1534	***	2.9152	***
*DGAP*^*(*+*)*^*.Centro*	3.3211	***	1.8959	***	1.1527	***	2.9257	***
*GAP.Centro*	0.8148	*	0.5779		0.6604		1.5792	
*L.Centro_HC*			-0.1232	***	0.0383		0.7869	***
*L.Centro_Y_1*	0.8172	***	0.8726	***	0.8959	***	0.5797	***
*RSE*	0.032		0.034		0.045		0.141	
*DGAP*^*(−)*^*.Lisboa*	2.6385	**	1.8251	**	1.4330	**	3.762	***
*DGAP*^*(*+*)*^*.Lisboa*	2.6552	**	1.8290	**	1.4580	**	3.857	***
*GAP.Lisboa*	0.0207		0.2986		-0.6172		-5.710	
*L.Lisboa_HC*			-0.1259	**	0.0785		1.831	***
*L.Lisboa_Y_1*	0.8598	***	0.8828	***	0.8728	***	0.339	***
*RSE*	0.031		0.04		0.038		0.182	
*DGAP*^*(−)*^*.Madeira*	3.233	***	1.7836	***	0.95014	**	0.6037	
*DGAP*^*(*+*)*^*.Madeira*	3.253	***	1.7713	***	1.00751	**	0.7400	
*GAP.Madeira*	0.404		1.6209	**	-0.41436		-3.1332	
*L.Madeira_HC*			-0.1875	***	-0.00146		1.0149	**
*L.Madeira_Y_1*	0.797	***	0.8701	***	0.90014	***	0.6656	***
*RSE*	0.047		0.044		0.057		0.269	
*DGAP*^*(−)*^*.Norte*	3.8491	**	1.9325	**	1.3081	**	3.4043	***
*DGAP*^*(*+*)*^*.Norte*	3.8361	**	1.9406	**	1.3136	**	3.3817	***
*GAP.Norte*	0.9960		-0.3194		0.1044		2.7949	*
*L.Norte_HC*			-0.0854	**	0.1234		1.0492	***
*L.Norte_Y_1*	0.7953	***	0.8691	***	0.8722	***	0.5101	***
*RSE*	0.039		0.04		0.053		0.173	

***, ** and * indicate statistical significance at, respectively, the 1, 5 and 10% significance levels. The results of the Wald test with H0: equality of the estimated coefficients of the dummies for negative and positive output gaps, indicate that: Models A1 and A4 -the null is not rejected for any region; Model A2: the null is rejected only for *Alentejo* (significance level = 4.1%) and *Açores* (7.6%); Model A3: the null is rejected for *Açores* (0.5%), *Lisboa* (9.6%) and *Madeira* (4.1%)

We next analyse the results for models A2, A3 and A4 presented in Table 2. For many of the regions and dependent variables, the estimated coefficients for the respective regional output gaps are negative but not statistically significant. This is the case of *Açores* (models A2 and A3), *Lisboa* (models A3 and A4), *Madeira* (models A3 and A4) and *Norte* (model A2). In the case of *Lisboa* the estimated coefficients is -5.7. If we multiply this number by the maximum values of the respective output gaps the result only reaches—0.095. The former is lower than 3.9, the value of the associated *DGAP*^*(−)*^ coefficient.

For the different regions, the estimated coefficient of human capital is always negative and statistically significant in the case of model A2, while in most regions it is positive for models A3 and A4 but only statistically significant in the latter. These findings suggest that higher availability of human capital in a region is associated with a decrease in employment of workers with lower schooling levels (G1). In the case of *Açores*, the sign of the estimated coefficient of human capital is also negative in model A3. In the case of model A2, according to the Wald test used it is not possible to reject the null hypothesis of equality of the estimated coefficients *DGAP*^*(*+*) *^and *DGAP*^*(−)*^ for *Alentejo* (significance level, 4.1%) and *Açores* (7.6%). For *Alentejo*, the *DGAP*^*(*+*)*^ estimate is lower than the *DGAP*^*(−)*^ estimate, but the difference is small (-0.025). For *Açores* the opposite applies but also in this case the value for the difference is small (+ 0.048). In face of these results, the evidence pointing to a different behaviour of the former two regions is thus not compelling. For instance, for *Alentejo* we have at most a tenuous indication of a jobless recovery situation. For the remaining five regions we cannot reject the null hypothesis of equality of *DGAP*^*(*+*)*^ and *DGAP*^*(−)*^ estimates and hence we can again conclude that there is labour market resilience for employees with less than 9 years of schooling.

The results for model A3 indicate that in the case of *Açores*, *Lisboa* and *Madeira* the null hypothesis of equality of the estimated coefficients for the positive and negative output gaps dummies is rejected at a significance level of 0.5%, 9.6% and 4.1%, respectively. Based on the respective level of significance (9.6%, close to 10%) we can ignore the case of *Lisboa*. The differences between the dummies’ coefficients are 0.060 and 0.057 for *Açores* and *Madeira*, respectively. For five regions, *Alentejo*, *Algarve*, *Centro*, *Lisboa* and *Norte*, the Wald test of the null hypothesis of equality of *DGAP*^*(*+*)*^ and *DGAP*^*(−)*^ estimates does not reject the null. Thus, the results indicate that the labour markets in these regions are resilient in terms of employees with medium human capital levels. As for *Açores* and *Madeira*, resilience cannot be confirmed but since in the case of these regions *DGAP*^*(*+*)*^ estimates are higher than the ones for *DGAP*^*(−)*^ the hypothesis of jobless recovery does not seem to apply. Concerning the results for model A4, they confirm the hypothesis of labour market resilience in all the NUTS2 regions for employees with high levels of human capital.

We now turn to the analysis of the findings concerning the effect of the PGR on employment (see Tables 3 and 4) based on the results of the estimation of our model B defined in Eq. (2). Table 3 contains the results for the regional SUR models considering the different measures of employment (HT, G1, G2 and G3) models B1, B2, B3 and B4 respectively. The results suggest that the effects of the PGR differ across regions. Additionally, the inclusion of the time dummy variables covering the period of the PGR alters the statistical quality of the previous models in some of the regions.

**Table 3:** SUR Results for the regional employment models with a focus on the PGR period (Model B)

	Model B1		Model B2		Model B3		Model B4	
*Dependent variable*	HT		G1		G2		G3	
*DGAP*^*(−)*^*.Alentejo*	1.88756	*	-0.37127		0.98887	**	2.72703	**
*DGAP*^*(*+*)*^*.AlentejoP*	1.86886	*	-0.41443		0.94755	**	2.82737	**
*GAP.Alentejo*	1.65054	**	2.73588	***	1.97518	***	-1.19645	
*L.Alentejo_HC*			-0.18533	***	-0.01232		0.92338	**
*L.Alentejo_Y_1*	0.88963	***	1.06802	***	0.91203	***	0.50122	***
*S_2009*	-0.03396		-0.00264		-0.00553		0.01698	
*S_2010*	-0.07169		-0.07194	**	-0.09111	**	-0.14739	
*S_2011*	-0.06265		-0.03349		-0.02278		-0.06769	
*S_2012*	-0.07674	*	-0.00959		-0.08560	**	-0.02507	
*S_2013*	-0.00423		0.06456		0.04157		0.00536	
*R_2014*	0.01829		0.09024	*	0.02973		0.08832	
*R_2015*	0.00329		0.06288		0.04212		0.02675	
*R_2016*	0.01831		0.07094		0.03825		0.13452	
*R_2017*	0.02223		0.06399		0.11833	***	0.06371	
*R_2018*	0.00844		0.06581		0.06427		0.06541	
*RSE*	0.038		0.024		0.031		0.204	
*DGAP*^*(−)*^*:Algarve*	0.58131		-0.15675		0.59208	**	0.9554	
*DGAP*^*(*+*)*^*.Algarve*	0.62312		-0.13997		0.62573	**	1.1555	
*GAP.Algarve*	-0.49701		0.45481		-0.47196		-2.8720	
*L.Algarve_HC*			-0.15301		-0.05563		1.4946	**
*L.Algarve_Y_1*	0.96679	***	1.04582	***	0.95842	***	0.5454	***
*S_2009*	-0.15189	***	-0.12516	***	-0.10806	***	-0.0684	
*S_2010*	-0.18509	***	-0.16428	***	-0.17830	***	-0.4630	*
*S_2011*	-0.06098	*	-0.04462		-0.00472		-0.1340	
*S_2012*	-0.15256	***	-0.09880	**	-0.13754	**	-0.2277	
*S_2013*	-0.05179		0.00161		-0.01967		-0.2135	
*R_2014*	0.01441		0.06391		0.05562		-0.1509	
*R_2015*	0.03649		0.07264		0.05341		-0.0710	
*R_2016*	0.00820		0.05162		0.04993		-0.2294	
*R_2017*	0.01143		0.02826		0.06014	*	-0.1343	
*R_2018*	-0.00416		0.02086		0.05464		-0.1369	
*RSE*	0.031		0.024		0.028		0.216	
*DGAP*^*(−)*^*:Açores*	-0.19834		0.08849		0.34731		2.76750	**
*DGAP*^*(*+*)*^*.Açores*	-0.19393		0.09623		0.45742		2.79435	**
*GAP.Açores*	-2.17879		-0.56902		-3.53921	*	3.71220	
*L.Açores_HC*			-0.05682		-0.14920		1.37545	**
*L.Açores_Y_1*	1.01543	***	1.00513	***	0.99857	***	0.29989	
*S_2009*	-0.06858		-0.02981		0.02196		0.16223	
*S_2010*	-0.13637	**	-0.12585	**	-0.12275	**	-0.09458	
*S_2011*	-0.07271		-0.08527		-0.03726		-0.00011	
*S_2012*	-0.13215	**	-0.09529		-0.04097		0.12283	
*S_2013*	-0.04744		-0.04819		-0.02651		0.10545	
*R_2014*	-0.13601	**	-0.03416		-0.02735		0.16115	
*R_2015*	-0.05749		-0.07683		0.03372		0.07017	
*R_2016*	0.03874		0.02285		0.01997		0.10116	
*R_2017*	0.01162		0.05494		-0.04475		0.18133	
*R_2018*	0.00263		0.00985		-0.00189		0.14388	
*RSE*	0.045		0.04		0.05		0.242	
*DGAP*^*(−)*^*:Centro*	1.253526	*	1.35289		1.19373	**	3.83912	***
*DGAP*^*(*+*)*^*.Centro*	1.257990	*	1.34725		1.15899	**	3.86126	***
*GAP.Centro*	0.442487		1.17158		2.48516	**	2.04368	
*L.Centro_HC*			-0.05216		-0.00905		1.03605	**
*L.Centro_Y_1*	0.932041	***	0.90429	***	0.90178	***	0.44338	***
*S_2009*	-0.06463	**	-0.05092		-0.01482		0.02451	
*S_2010*	-0.06104	**	-0.08840	**	-0.05528		-0.12126	
*S_2011*	-0.05099	**	-0.05938		0.00369		-0.06324	
*S_2012*	-0.08938	***	-0.08870	**	-0.08061	**	-0.02305	
*S_2013*	-0.03134		-0.03385		0.02645		0.00652	
*R_2014*	0.00676		0.00212		0.08151	*	0.04384	
*R_2015*	-0.00025		-0.02313		0.04190		0.04477	
*R_2016*	0.01021		-0.01435		0.05269		0.06397	
*R_2017*	0.01318		-0.00672		0.07219	*	0.05716	
*R_2018*	0.00380		-0.02506		0.08436	**	0.08216	
*RSE*	0.019		0.026		0.032		0.206	
*DGAP*^*(−)*^*:Lisboa*	-0.00675		5.8558	**	1.42630	*	4.5613	***
*DGAP*^*(*+*)*^*.Lisboa*	-0.00294		5.8744	**	1.43490	*	4.6521	***
*GAP.Lisboa*	0.76423	*	-0.5971		-0.02733		-8.5128	
*L.Lisboa_HC*			0.1185	*	0.13713		2.2212	***
*L.Lisboa_Y_1*	1.00163	***	0.5396	**	0.86394	***	0.1977	
*S_2009*	-0.06450	***	-0.0633	**	-0.05109		0.0265	
*S_2010*	-0.09342	***	-0.1460	***	-0.09208	**	0.0372	
*S_2011*	-0.04913	**	-0.1264	***	-0.02909		0.0527	
*S_2012*	-0.08472	***	-0.2063	***	-0.10387	***	-0.1017	
*S_2013*	-0.02724		-0.1885	**	-0.02511		-0.0711	
*R_2014*	0.00736		-0.1650	**	0.00019		-0.0567	
*R_2015*	0.00503		-0.1701	**	-0.00574		-0.0296	
*R_2016*	0.02384		-0.1479	*	0.02198		-0.0179	
*R_2017*	0.00911		-0.1501	**	0.02445		0.0472	
*R_2018*	0.00483		-0.1632	**	0.02590		0.0775	
*RSE*	0.016		0.018		0.029		0.256	
*DGAP*^*(−)*^*:Madeira*	1.4317	**	-0.0089		0.85080	**	0.82146	
*DGAP*^*(*+*)*^*.Madeira*	1.4077	**	-0.0699		0.91411	**	0.92119	
*GAP.Madeira*	0.8607		2.7383	**	-2.06805		3.62504	
*L.Madeira_HC*			-0.1695		0.08760		1.02280	
*L.Madeira_Y_1*	0.9132	***	1.0392	***	0.89145	***	0.63978	***
*S_2009*	-0.0779	**	-0.0595		-0.02279		0.10701	
*S_2010*	-0.0887	***	-0.0814	*	-0.14743	***	-0.14199	
*S_2011*	-0.0711	**	-0.0788		-0.01926		-0.17248	
*S_2012*	-0.1327	***	-0.0528		-0.16249	***	0.12344	
*S_2013*	-0.1219	**	-0.0560		-0.13378	**	0.06613	
*R_2014*	-0.0442		0.0434		-0.03974		0.05963	
*R_2015*	-0.0328		0.0446		0.01485		0.07028	
*R_2016*	-0.0431		0.0358		0.00863		0.00378	
*R_2017*	0.0114		0.0406		0.05438		-0.10703	
*R_2018*	0.0313		0.0786		0.04600		-0.01466	
*RSE*	0.026		0.037		0.032		0.369	
*DGAP*^*(−)*^*.Norte*	0.41995		9.8896	***	1.8366	**	4.85350	***
*DGAP*^*(*+*)*^*.Norte*	0.39927		9.8925	***	1.8425	**	4.78424	***
*GAP.Norte*	0.45819		-0.1718		-0.1981		4.49119	*
*L.Norte_HC*			0.1674	**	0.2046		1.38315	**
*L.Norte_Y_1*	0.97911	***	0.2435		0.8128	***	0.31974	*
*S_2009*	-0.09067	*	-0.0966	**	-0.0611		-0.00241	
*S_2010*	-0.03976		-0.1627	***	-0.0729		-0.06610	
*S_2011*	-0.04043		-0.1931	***	-0.0365		-0.02185	
*S_2012*	-0.08679	**	-0.2610	***	-0.1130	**	-0.01509	
*S_2013*	-0.02377		-0.2614	***	-0.0159		0.01041	
*R_2014*	0.00043		-0.2509	***	0.0201		0.03146	
*R_2015*	0.01155		-0.2434	**	0.0461		0.05513	
*R_2016*	0.01049		-0.2407	**	0.0570		0.07943	
*R_2017*	0.03159		-0.2295	**	0.0749		0.14774	
*R_2018*	0.02911		-0.2297	**	0.0967	*	0.18253	
*RSE*	0.036		0.032		0.04		0.255	

***, ** and * indicate statistical significance at, respectively, the 1, 5 and 10% significance levels. The results of the Wald test with H0: equality of the estimated coefficients of the dummies for negative and positive output gaps, indicate that: Model B1—does not reject the null except for *Algarve*, at a significance level of 2.5%; Model B2—does not reject the null except for *Alentejo*, at a significance level of 0.1% and *Madeira* at a significance level 7.7%; Model B3—rejects the null for *Alentejo* (0.001%), *Algarve* (0.5%), *Açores* (1%) and *Madeira* (7.3%), for the other regions the null is not rejected; Model B4 -rejects the null only for *Algarve* (4.8%); for the other regions equality the null is not rejected

**Table 4:** Results of the test for labour market resilience to the PGR

	HT		G1		G2		G3	
	Sum(S + R)	PL	Sum(S + R)	PL	Sum(S + R)	PL	Sum(S + R)	PL
*Alentejo*	-0.1787		0.04962		0.05267		1.019	
*Algarve*	-0.5359	***	-0.3678		-0.2848		-0.197	
*Açores*	-0.5978	***	-0.4744		-0.3731		2.185	
*Centro*	-0.2637	***	-0.4155		0.1187		1.069	
*Lisboa*	-0.2689	***	-1.245	***	-0.1232		2.108	
*Madeira*	-0.5696	***	-0.3337		-0.36		1.032	
*Norte*	-0.1983		-1.772	***	0.1033		1.062	

H0: the sum of the coefficients of the S and R dummy variables is equal to zero. ***—significance level 1%; probability level (PL) associated with the Chi-squared statistic for a Wald test

In what follows we focus on the additional information provided by the estimation of model B relative to the results for model A. Concerning the results for model B1, only for *Algarve* is the null hypothesis that the sum of the coefficients of the dummy variables for the years corresponding to the PGR rejected (see Table 4). However, the robustness of this conclusion is hindered by the non-rejection of the null hypothesis for the estimated coefficient of the output gap and of equality of *DGAP*^*(*+*)*^ and *DGAP*^*(−)*^ estimates. Thus, we can maintain the non-rejection of the business cycle effect on employment with positive effects compensating the negative effects in terms of total hours worked. Labour market resilience is thus confirmed for all regions. Regarding the coefficients of the PGR time dummies, their sum is always negative (first column, Table 4), although for *Alentejo* and *Norte* we cannot reject the hypothesis that the respective sum is zero.

The conclusions are however different when looking at the results for models B2, B3 and B4. In the case of model B2, there is no compensation in terms of *DGAP*^*(*+*)*^ and *DGAP*^*(−)*^ estimates effects on employment in *Alentejo* (significance level: 0.1%) and *Madeira* (7.7%) since they are different according to the results of the Wald test. But looking at the values for the estimated coefficients individually, in those two regions it is not possible to reject the null hypothesis that they are each equal to zero. Thus, with the exception of *Alentejo*, we confirm the hypothesis that opposite signed cyclical components of output have a symmetric impact on the level of employment of the less educated employees and consequently we confirm the labour market resilience hypothesis.

With respect to the PGR time dummy variables (Table 4), the null hypothesis that the sum of the respective coefficients is equal to zero is rejected only for the regions of *Lisboa* and *Norte*. Since the sign of the sum is negative these results suggest that employment of less educated employees has not yet recovered from the negative effects of the PGR. As for Model B3, there is no compensation for *Alentejo* (0.001%), *Algarve* (0.5%), *Açores* (1%) and *Madeira* (7.3%). In *Alentejo*, *Algarve* and *Madeira* the null hypothesis for the equality of *DGAP*^*(*+*)*^ and *DGAP*^*(−)*^ estimates is rejected and a higher value for the coefficient of the negative gap was obtained for *Alentejo.* The opposite applies to *Algarve* and *Madeira*, suggesting that *Alentejo* is undergoing a jobless recovery and that in the other two regions the results do not support the hypothesis of labour market resilience.

As far as the effects of the PGR are concerned, according to the results presented in Table 4 the hypothesis that the sum of the coefficients of the different time dummies is equal to zero is rejected for all the regions. In any case, the respective sum is in most cases negative, with the exception of *Alentejo* and *Norte*, for which it is positive. This result confirms the previous finding for employees with at least 9 and less than 12 years of schooling (see model A3, Table 2) according to which the cyclical compensation hypothesis applies to all the business cycles covered in the period under analysis, including the recent PGR period.

Finally, the results for model B4 that considers as dependent variable employees with more than 12 years of schooling indicate that the business cycle compensation hypothesis corresponding to the equality of *DGAP*^*(*+*)*^ and *DGAP*^*(−)*^ estimates is confirmed in all regions except *Algarve*. However, it is also not possible to reject the null of the *DGAP*^*(*+*)*^ and *DGAP*^*(−)*^ estimates in the case of Algarve and at the same time the null for each of the time dummies corresponding to the PGR variables is also never rejected (Table 4). The evidence found thus supports the business cycle compensation hypothesis for the employees with more than 12 years of schooling in all the regions, and this conclusion extends to the specific effects of the PRG.

Naturally, a comparison between the main findings in our study with those in the literature reviewed in Sect. 2 is in order, albeit this is only possible taking a quite broad picture of the results. Our findings suggest that: i) the evidence supporting the existence of labour market resilience across the seven Portuguese NUTS-2 regions is mixed, in line with the results of studies applied to other regions such as Fingleton et al. (2012) and Martin and Gardiner (2019); ii) similar to previous studies we confirm the importance of human capital to labour market resilience after severe adverse shocks, see e.g. Di Caro (2018)); Giannakis and Bruggeman (2017); Kitsos and Bishop (2018); Hennebry (2020), also associated with, so far, a situation of jobless recovery, a consequence of the negative impact of the PGR on employment of workers less endowed with human capital. In this respect, Fingleton et al. (2012) find similar results for overall employment relative to major shocks that hit the UK economy over the period 1971–2010.

## Conclusion

In this study we examined the importance of human capital for labour market resilience in a sample of seven Portuguese NUTS2 regions observed over the period 1995–2018. We found that for the whole period under analysis, characterized by different regional business cycles and thus shocks, all regions were able to recover in terms of the different employment measures considered, total hours worked and employees with low, medium and high levels of education. Irrespective of the time that recovery takes and region, overall the effects of shocks on regional employment have been merely temporary. However, severe disruptions such as that occurred in 2007–8 seem to be producing differentiated regional effects and human capital makes a difference for regional employment resilience when the shock is particularly severe. The former conclusion stems from our analysis of the differentiated impact of the Portuguese Great Recession (PGR). In this respect, our findings indicate no resilience in terms of total hours worked and employment of workers with low levels of education, corresponding so far to a situation of jobless economic recovery. The conclusions are mixed for employment of workers with medium levels of education, while we found evidence of labour market resilience to the PGR for employment of workers with high levels of education.

Differently from previous contributions, our study pays attention to the specificities of regional business cycles and provides a framework to characterize resilience to several shocks (determined by the regional data) over an extended period of analysis. At the same time, this approach accommodates the analysis of the possible differentiated effects of specific shocks and allows for the analysis of the importance of certain factors for regional resilience. For instance, similar to what we did for human capital, looking at employment of workers with different levels of education, it is possible to consider as dependent variable employment in different sectors of activity and in this way investigate in a straightforward way the importance of economic structure for regional resilience.

What course of policy action does the evidence found suggest? The higher resilience of highly educated employees to the severe adverse shock that resulted in the Portuguese Great Recession (2009–2014) advises the implementation of policies that improve the quantity of education, possibly enabling universal access to tertiary education. This would make all regions more resilient and probably result in a more equal country at the regional level. Regional policies aligned with the objectives of decarbonisation and digital transition aimed at promoting regional productive structures based on sectors with higher demand for skilled workers may also strengthen labour market resilience to adverse shocks through human capital. Our findings also highlight the importance of education policy to promote labour market resilience at the regional level in the aftermath of the new SARS-CoV-2 coronavirus and COVID-19 disease crisis period initiated in the first quarter of the year 2020, which is undermining the capacity of the education system to promote human capital accumulation. Regarding human capital, Houston (2020) stresses that similar to “normal” recessions one major source of resilience to the Covid-19 recession is a highly skilled workforce to protect against unemployment.

The extent and duration of the pandemic shock with its sudden and widespread restrictions that impacted regions differently will likely have specific effects on regional labour market resilience and demands continuous monitoring of the ability of regions to resist and recover from this unparalleled (at least in the twenty-first century) adverse shock. In this respect and for the case of Portugal, Carvalho et al. (2022), using monthly data on electronic payments between the months of January 2017 and August 2020, show that the impact of this shock was so far very unequal for the universe of 308 municipalities in Portugal. For the case of Chinese cities, Hu et al. (2022) conclude that there are significant differences in terms of regional economic impact between COVID-19 and the 2008 financial crisis. Brada et al. (2021), using the pattern of recovery from the financial crisis of NUTS-3 regions in Central and East European (CEE) countries’ to simulate recovery from the shock to employment caused by the COVID-19 pandemic, forecast that very few regions will have recovered pre-pandemic levels of employment even two years after the start of recovery.

This study has some limitations that represent avenues for future research. We focus on the recovery aspect of labour market resilience, but we do not analyse resistance, the sensitivity of the region to the recessionary shock, nor do we detail the analysis of the speed of recovery. Also, the aspects of reorientation and renewal in regional economic resilience are not addressed. We leave also to future research the investigation of other potential determinants of labour market resilience in the Portuguese regions. Our findings are suggestive of the importance of human capital and the severity of shocks for regional employment resilience but a deeper understanding of the importance of these features for resilience requires more sophisticated statistical techniques, which are however more demanding in terms of data and assumptions. The SUR specification applied does not take into consideration that shocks to one region may spill over through time to other regions and it also does not deal with omitted- variable bias nor the possibility of reverse causality. Dynamic panel models with spatial effects are a possible future research avenue to deal with the former issues.

## Data Availability

The data used in this study was collected from publicly available sources.

## Footnotes

1 According to Martin and Gardiner (2019), while a search in the Web of Science in the scientific fields of environmental studies, business and management studies, planning, urban studies, economics and economic geography retrieves around 230 studies that mention “resilience” in the respective title published in 2007, the same search for the year 2017 identifies more than 1,200 works.
